# Organbezogene Folgeerscheinungen von COVID‑19 bei Erwachsenen

**DOI:** 10.1007/s00103-022-03513-2

**Published:** 2022-03-16

**Authors:** Jan K. Hennigs, Tim Oqueka, Lars Harbaum, Hans Klose

**Affiliations:** 1 Abteilung für Pneumologie, Universitätsklinikum Hamburg-Eppendorf, Martinistr. 52, 20246 Hamburg, Deutschland; 2 II. Medizinische Klinik und Poliklinik, Universitätsklinikum Hamburg-Eppendorf, Hamburg, Deutschland

**Keywords:** Long Haulers, Folgen, Long-COVID, Post-COVID, Symptome, Long Haulers, Sequelae, Long COVID, Post-COVID syndrome, Symptoms

## Abstract

Organbezogene Folgeerscheinungen nach COVID-19 sind häufig und vielgestaltig. Ab 4 Wochen nach Akutinfektion mit SARS-CoV‑2 werden sie unter dem Begriff „Long-COVID“ zusammengefasst.

Nach schweren Akutverläufen treten organbezogene Folgeerscheinungen häufiger auf. Dauer und Intensität variieren jedoch interindividuell stark. Die SARS-CoV-2-Spezifität der Folgeerscheinungen ist ebenfalls weiter unklar. Während sich in der Frühphase nach schweren Verläufen zumeist pulmonale Folgeerscheinungen einstellen, müssen diese nicht auf die Lunge begrenzt bleiben, sondern können prinzipiell jedes Organ betreffen. Die adäquate Diagnostik von COVID-19-Folgeerscheinungen stellt daher eine interdisziplinäre Herausforderung dar. Auch die Therapie richtet sich nach Art, Umfang und Ursache der jeweiligen Folgeerscheinung. Allgemeinmedikamentöse oder zielgerichtete Therapieoptionen gegen Long-COVID bestehen bisher nicht.

Im vorliegenden Übersichtsartikel berichten wir über Häufigkeit, Dauer, Spezifität sowie Art und Umfang organspezifischer COVID-19-Folgeerscheinungen und geben einen Überblick über diagnostisches und therapeutisches Vorgehen (mit Datenstand November 2021).

## Abstract (en)

Organ-specific sequelae after COVID-19 occur frequently and are highly diverse in their features. Sequelae and symptoms persisting for more than four weeks after COVID-19 define the condition “long COVID.”

Organ-specific sequelae of COVID-19 generally occur more often after severe disease. Yet, duration and intensity of organ-specific sequelae are highly variable. While pulmonary sequelae typically persist after more severe acute disease, COVID-19 sequelae may also develop weeks after infection and can affect any organ. The degree of SARS-CoV‑2 specificity of COVID-19 sequelae, however, remains unclear. Thus, diagnosis and treatment of COVID-19 sequelae represent an interdisciplinary challenge. Diagnostic and therapeutic approaches are guided by type, extent, and cause of the specific sequelae as targeted therapy options for long COVID are lacking.

In the present work, we review current knowledge regarding the prevalence/incidence, duration, specificity, type, and extent of organ-specific COVID-19 sequelae and summarize current diagnostic and therapeutic strategies (as of November 2021).

## Einführung

Seit Ende Dezember 2019 breiten sich Infektionen durch das Severe Acute Respiratory Syndrome Coronavirus 2 (SARS-CoV-2) als Coronaviruskrankheit (COVID-19) pandemisch aus. Obwohl häufig primär die Lunge betroffen ist, stellt COVID-19 eine Systemkrankheit dar [1]. Mit wachsendem Verständnis der Erkrankung zeigt sich, dass neben der Akutproblematik auch länger anhaltende Folgeerscheinungen nach COVID-19 sehr häufig auftreten [2].

Halten diese länger als 4 Wochen nach der Akutinfektion an, spricht man von „Long-COVID“. Das „Post-COVID-Syndrom“ umfasst alle Folgeerscheinungen, die länger als 12 Wochen nach der Akutinfektion noch vorliegen oder neu hinzugekommen sind [3]. Das genaue Verständnis der COVID-19-Folgeerscheinungen ist essenziell, um Ressourcen des Gesundheitssystems adäquat bereitstellen und weitere wissenschaftliche Schwerpunkte identifizieren zu können.

Die am häufigsten genannte COVID-19-Folgeerscheinung ist Fatigue, d. h. anhaltende Erschöpfungszustände, die sich oftmals nicht einer eindeutig kausalen Organdysfunktion zuordnen lassen. Häufig treten jedoch auch organbezogene Folgeerscheinungen wie Belastungseinschränkung, Dyspnoe, thorakale oder Muskelschmerzen bis hin zu Haarausfall und anhaltenden Geruchs- und Geschmacksstörungen auf [2, 4]. Während die psychischen Folgeerscheinungen meist unabhängig vom Schweregrad der Akuterkrankung sind, scheinen organbezogene Folgeerscheinungen häufiger bei Patient*innen mit schwerem COVID-19-Akutverlauf aufzutreten. Die Diagnostik von organbezogenen Ursachen funktioneller Beschwerden ist zudem vielfach nicht trivial und erfordert daher ein systematisches, interdisziplinäres Vorgehen. Verkomplizierend hierzu sind Patient*innen mit Risikofaktoren für einen schweren COVID-19-Verlauf häufig vorerkrankt. Die Symptome der Vorerkrankung überlappen häufig mit Symptomen von COVID-19-Folgeerscheinungen, was die Abgrenzung COVID-19-spezifischer Folgen von einer Verschlechterung (Exazerbation) der Grunderkrankung erschwert. Zudem ist unklar, in welchem Ausmaß Folgeerscheinungen spezifisch für COVID-19 sind oder Ausdruck bzw. Folge eines schweren generalisierten Virusinfekts [3].

Dieser Übersichtsartikel versucht daher Häufigkeit, Dauer, Spezifität sowie Art und Umfang organspezifischer COVID-19-Folgeerscheinungen zusammenzufassen. Nicht beschrieben werden Folgeerscheinungen aufgrund von Störungen im Bereich der vegetativen Regulation oder von Biomediatoren. Abschließend bietet der vorliegende Aufsatz einen Überblick über die zum Zeitpunkt November 2021 noch sehr limitierten therapeutischen Optionen sowie ein Beispiel für strukturierte Differenzialdiagnostik bei COVID-19-Folgeerscheinungen.

## Begriffsdefinition von COVID-19-Folgeerscheinungen

**Figure 1:**
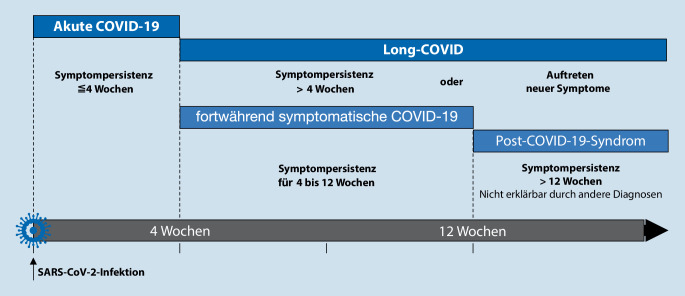

Bisher gab es keine einheitliche Nomenklatur hinsichtlich COVID-19-Folgeerscheinungen, die jedoch allein aufgrund der sehr dynamischen Datenlage notwendig ist. Die deutsche, interdisziplinäre S1-Leitlinie „Post-COVID/Long-COVID“ [3] bietet hier mit einer zeitlichen Einordnung der Folgeerscheinungen einen Definitionsversuch, der sich auch an internationalen Konsortien (z. B. des National Institute for Health and Care Excellence – NICE) orientiert [5]. Demzufolge fallen unter den Begriff „Long-COVID“ alle Symptome, die länger als 4 Wochen nach der Akutinfektion persistieren. Erst wenn nach über 12 Wochen persistierende oder neu aufgetretene COVID-19-assoziierte Symptome bestehen, wird von einem „Post-COVID-Syndrom“ gesprochen, da postinfektiöse Allgemeinbeschwerden bis zu 3 Monate nach Atemwegsinfekten anhalten können. Abb. 1 gibt einen zusammenfassenden Überblick über die vorgeschlagene Nomenklatur.

Da in der Literatur verschiedene Termini zur Beschreibung ähnlicher Phänomene verwendet werden – so werden beispielsweise die Begriffe „Sequelae“, „Persistent Symptoms“, „Long Haulers“, „Post Acute COVID Syndrome“, „Long COVID“ und „Post COVID“ häufig synonym verwendet –, ist eine Harmonisierung der Begrifflichkeiten von größter Wichtigkeit. Im Alltag sollte deswegen diesbezüglich den Empfehlungen aus der S1-Leitlinie und von NICE gefolgt werden.

## Prävalenz und Dauer von COVID-19-Folgeerscheinungen

Eine genaue Abschätzung der Häufigkeit von somatischen bzw. organbezogenen COVID-19-Folgeerscheinungen ist schwierig, insbesondere deshalb, da die zum Thema veröffentlichten Arbeiten häufig nicht direkt miteinander vergleichbar sind: Zum einen erschwert eine uneinheitliche Terminologie die Vergleichbarkeit, zum anderen weisen die Studien eine immense Heterogenität hinsichtlich des Schweregrads der initialen Erkrankung, des Nachbeobachtungszeitpunkts und der Studiengröße auf. Weiterer Bias wird durch unterschiedliche Rekrutierungsmethodik der Kollektive eingeführt [6].

Dadurch schwanken je nach Studie die Angaben zur Prävalenz von persistierenden Beschwerden stark und liegen so beispielsweise binnen 8 Wochen nach COVID-19 zwischen 4,5 % [7] und 89 % [8]. Basierend auf einer aktuellen Metaanalyse wiesen von 47.910 Erwachsenen nach mildem bis schwerem COVID-19-Verlauf 80 % der Infizierten im Zeitfenster 2 Wochen bis 3 Monate später Folgeerscheinungen auf [4].

In einer prospektiven, kontrollierten Studie, in der SARS-CoV-2-positive und SARS-CoV-2-negative Teilnehmer*innen mit Infektsymptomen aus den USA, dem Vereinigten Königreich und Schweden hinsichtlich Alter, Geschlecht und Body-Mass-Index gematcht waren, gaben 13,3 % der 4182 inzidenten COVID-19-Fälle per Selbstauskunfts-App (von insgesamt 4,3 Mio. Teilnehmer*innen) über mindestens 28 Tage persistierende Symptome an, während nach 8 Wochen 4,5 % und nach 12 Wochen noch 2,3 % der COVID-19-Fälle persistierende Beschwerden berichteten [7]. Ähnliche Daten liefert das britische „Office for National Statistics“ [9]: Von 26.147 PCR-positiven COVID-19-Patient*innen aus dem britischen *Coronavirus Infection Survey* zeigten 3 % der Teilnehmenden länger als 12 Wochen und 1,3 % länger als 18 Wochen andauernde Symptome, während in der PCR-negativen Kontrollgruppe mit Atemwegsinfekt 0,5 % bzw. 0,2 % der Teilnehmenden länger als 12 bzw. 18 Wochen anhaltende Symptome berichteten [9]. Demgegenüber stehen Daten aus Deutschland von Augustin und Kollegen, die in einer prospektiven Kohorte von 442 COVID-19-Patient*innen mit initial mildem Verlauf bei 34,8 % der Patient*innen über 7 Monate anhaltende Beschwerden detektierten [10].

Die Symptomdauer nach COVID-19 liegt auf der Basis beider o. g. Kollektive zwischen 11 Tagen (IQR: 6–19 Tage) im Median [7] und 39,5 Tagen (95 % Konfidenzintervall (KI) 38,5–42,5 Tage, [9]) im Mittel. Beide Studien zeigen (im Gegensatz zur deutschen Kohorte) eine deutliche Abnahme der Symptomhäufigkeit über die Zeit.

## Schweregradeinteilung der funktionellen Einschränkungen nach COVID-19

Zur Abschätzung von COVID-19-assoziierten oder koinzidentellen Organschädigungen im postakuten Verlauf stehen bisher nur COVID-19-unabhängige, etablierte Biomarker (z. B. Lungenfunktionsprüfung, Echokardiografie, MRT, Troponin I/T, proBNP oder Kreatininwert/glomeruläre Filtrationsrate) zur Verfügung. Spezifische Post-COVID-19-Seromarker, die mit dem Schweregrad der Folgeerscheinungen korrelieren, sind bisher nicht identifiziert. Zur besseren Erfassung allgemeinfunktioneller Einschränkungen durch organbezogene und psychische COVID-19-Folgeerscheinungen im Alltag etablierten [11] und validierten [12] Klok und Kollegen die „Post-COVID-Functional-Status“(PCFS-)Skala. Die PCFS-Skala ist ein einfach einzusetzendes Instrument, dessen Einsatz auch von der deutschen Post-COVID-Leitlinie empfohlen wird [3].

## Risikofaktoren/Prädiktoren für das Auftreten von COVID-19-Folgeerscheinungen

Risikofaktoren bzw. Prädiktoren für das Auftreten von COVID-19-Folgeerscheinungen konnten in kürzlich veröffentlichten oder erst vorveröffentlichten, größeren Kohortenstudien identifiziert werden. Demnach sind Frauen bis zu 5‑mal häufiger von Folgeerscheinungen betroffen als Männer ([7], Odds Ratio (OR) = 5,09 (1,64–15,74), [13]; OR: 1,65 (1,26–2,17), [14]; OR: 1,51 (1,46–1,55), [15]), wobei sich dieser Unterschied im Alter (> 50 Jahre [13] bzw. > 70 Jahre [7]) nivelliert. Im Vergleich zu 18- bis 30-Jährigen steigt mit jeder Lebensdekade das Risiko für länger als 2 Monate anhaltende Folgeerscheinungen an (Frauen: OR: 2,19–8,61, Männer: 4,12–18,56, [7]). Eine ausgeprägt symptomatische Akuterkrankung (definiert als Auftreten von 5 oder mehr Akutsymptomen) wurde in 2 voneinander unabhängigen Arbeiten ebenfalls als Prädiktor für länger als 4 Wochen (OR: 3,95 (3,1–5,04), [7]) bzw. 6 Monate (OR: 2,69 (2,25–3,21), [14]) persistierende COVID-19-Folgeerscheinungen identifiziert. Auch wenn Patient*innen mit mildem Verlauf einer Akutinfektion ebenfalls in relevantem Ausmaß COVID-19-Folgeerscheinungen entwickeln können [16], waren Hospitalisation (OR: 1,62 (1,21–2,25), [14]) und insbesondere die Notwendigkeit einer intensivmedizinischen Betreuung (OR: 4,0 (2,66–6,02),[14]) mit mechanischer Beatmung (OR: 3,67 (1,61–8,38), [13]) mit einem deutlich erhöhten Risiko für Folgeerscheinungen assoziiert. Hinsichtlich organbezogener Vorerkrankungen ist bei beiden biologischen Geschlechtern gemeinsam bisher nur Asthma bronchiale als prädiktiv für länger als 2 Monate anhaltende COVID-19-Folgeerscheinungen beschrieben worden (OR: 2,14 (1,55–2,96), [7]), während bei Frauen auch das Vorhandensein kardiovaskulärer Risikofaktoren (CVRF) mit einem erhöhtem Risiko von über 6 Monate anhaltenden Folgeerscheinungen assoziiert ist (OR: 1,39 (1,03–1,89), [14]). Beachtenswert ist jedoch, dass sowohl Asthma als auch CVRF unabhängig von COVID-19 infektgetriggert typische Long-COVID-Symptome wie Belastungsintoleranz, Atemnot, Husten oder thorakale Schmerzen auslösen können.

## Organspezifische COVID-19-Folgeerscheinungen

Die genaue Angabe der Häufigkeit und Dauer organspezifischer COVID-19-Folgeerscheinungen ist aufgrund des heterogenen Studiendesigns der verfügbaren Arbeiten schwierig. Neben Fatigue, als häufigste Folgeerscheinung (s. Beitrag von Haller et al. in diesem Themenheft), schildern Patient*innen insgesamt besonders häufig anhaltende Belastungsintoleranz und (Belastungs‑)Dyspnoe als COVID-19-Folgeerscheinungen aus dem pulmonalen und kardialen Bereich. Dazu kommen ebenfalls sehr häufig persistierende Riech- und Geschmacksstörungen und Kopfschmerzen. Wiederholt werden zudem Reizhusten, thorakale und allgemeine Schmerzen, passagerer Haarausfall sowie Wortfindungsstörung und andere kognitive Störungen berichtet. Weniger häufig werden persistierende gastrointestinale Folgeerscheinungen, Schwindel, Palpitationen, Tachykardie, Sensibilitätsstörungen oder motorische Störungen angegeben. Insgesamt wurden mindestens 55 unterschiedliche Folgeerscheinungen identifiziert, darunter 42 somatische Beschwerden [4]. Tab. 1 zeigt, analog der deutschen S1-Leitlinie [3, 6], eine kategorische Einteilung der relevantesten organbezogenen COVID-19-Folgeerscheinungen nach der relativen Häufigkeit in der Literatur.

**Table 1:** 

Sehr häufig (> 20 %)	Häufig (20–10 %)	Weniger häufig (< 10 %)
Cor/Pulmo:	Cor/Pulmo:	Cor/Pulmo:
*Belastungsintoleranz*	*Husten*	*Auswurf*
*Dyspnoe*	*Palpitationen*	*Herzrhythmusstörungen*
	*Pectanginöse Beschwerden*	*Arterielle Hypertonie*
Dermis/muskuloskelettal:	*Tachykardien*	*Periphere Ödeme*
*Haarausfall*		
*Schmerzsyndrome*	Dermis/muskuloskelettal:	Gastrointestinaltrakt/Endokrinium:
	*Gelenkbeschwerden*	*Diarrhö*
HNO/Neuro:	*Hautveränderungen*	*Flush-Symptomatik*
*Geruchsstörungen*	*Hyperhidrosis*	
*Geschmacksstörungen*		HNO/Neuro:
	Gastrointestinaltrakt/Endokrinium:	*Lähmungen*
	*Übelkeit/Erbrechen*	*Sensibilitätsstörungen*
	*Verdauungsstörungen*	*Ohrenschmerzen*
	*Gewichtsschwankungen*	
		Inflammation:
	Inflammation:	*Schüttelfrost*
	*Intermittierendes Fieber*	

*HNO* Hals-Nasen-Ohren-Bereich

Die klinisch relevantesten organbezogenen Folgeerscheinungen werden im Folgenden, nach Organsystem sortiert, kurz erläutert. Einordnend muss jedoch erwähnt werden, dass in den meisten untersuchten Kollektiven in der Regel vorbestehende Organpathologien aufgrund fehlender Vordaten nicht ausgeschlossen werden konnten, sodass eine Überschätzung von Häufigkeit oder Schweregrad der Folgeerscheinungen möglich wäre. Generell scheint für alle organbezogenen COVID-19-Folgeerscheinungen mit Ausnahme dermatologischer Auffälligkeiten zu gelten, dass das Risiko des Auftretens innerhalb der ersten 6 Monate nach COVID-19 mit zunehmender, initialer Krankheitsschwere ansteigt [16]. Die pathogenetischen Zusammenhänge sind weiter größtenteils unklar und deren Klärung bedarf dringend weiterer Forschung.

### Pulmonale Folgeerscheinungen

Pulmonale Folgeerscheinungen von COVID-19 wurden bereits früh in vorwiegend kleineren Kollektiven als häufige Residuen berichtet (bis zu 44 % [8]). Hierbei zeigen sich in der Frühphase nach COVID-19 (0–3 Monate) hauptsächlich Einschränkungen der Lungenfunktion und Diffusionskapazität sowie radiologische Veränderungen in Form von „Milchglasinfiltraten“, Konsolidierungen und einem breiten Spektrum fibrotisch-narbigen Umbaus; deutlich seltener treten pulmonal-vaskuläre Komplikationen auf (z. B. Lungenarterienembolien; [17–21]).

In einer monozentrischen Querschnittsstudie aus Italien wiesen 4 Monate nach COVID-19 noch 51,6 % der untersuchten Fälle (113/219) eine Einschränkung der Diffusionskapazität (DLCO) < 80 % bzw. in 15,5 % der Fälle (34/219) < 60 % des Solls auf. Eine intensivmedizinische Behandlung war stärkster Prädiktor für eine DLCO < 60 % (OR 4,60 (1,85–11,48)). Weitere Lungenfunktionseinschränkungen wurden in diesem Kollektiv nicht berichtet [22].

In der Wuhan-Querschnittsstudie gaben 24–36 % der 1739 der initial aufgrund von COVID-19 hospitalisierten Patient*innen 6 Monate später persistierende Dyspnoe an. Eine Lungenfunktionsprüfung erfolgte bei 349 Patient*innen. DLCO-Einschränkungen < 80 % zeigten hierbei, abhängig vom initialen COVID-19-Schweregrad, 22–54 % der Fälle (OR 4,60 (1,85–11,48), schwer/kritisch vs. moderat Erkrankte). 2– 8 % der Fälle wiesen einen eingeschränkten Tiffeneau-Quotienten (FEV1/FVC = Einsekundenkapazität/forcierte Vitalkapazität) < 70 % als Ausdruck einer obstruktiven Ventilationsstörung auf, während die totale Lungenkapazität (TLC) als Marker einer restriktiven Ventilationsstörung bei 11–35 % abhängig vom initialen Schweregrad der Erkrankung eingeschränkt war (TLC < 80 %). Auffälligkeiten in der thorakalen Computertomografie wiesen 52–54 % der 353 untersuchten Fälle auf, wovon potenziell reversible Milchglastrübungen mit 41–48 % das prädominante radiologische Phänomen darstellten und potenziell irreversible, narbige Veränderungen selten (0–6 %) detektiert wurden [23].

Caruso et al. berichten dagegen in einer prospektiven Beobachtungsstudie an vorwiegend persistierend symptomatischen Patient*innen (77 % des Kollektivs), dass 6 Monate nach akuter, hospitalisierungspflichtiger COVID-19-Pneumonie 85/118 Fälle (72 %) weiterhin radiografische Zeichen einer interstitiellen Lungenerkrankung aufwiesen [24].

Eine weitere prospektive Analyse zeigt, dass 4 Wochen nach Entlassung bei 39 % (325/837) der Patient*innen nach SARS-CoV-2-Pneumonitis noch pulmonale Residuen bestehen. Zeichen einer interstitiellen Lungenerkrankung zeigten dabei allerdings nur 35/837 Patient*innen (4,8 %; [25]). Diese Daten decken sich mit Ergebnissen aus einer multizentrischen, prospektiven Studie von 175 wegen COVID-19 hospitalisierten Teilnehmenden, bei denen sich Symptomlast, lungenfunktionelle Veränderungen und radiografische Veränderungen im Verlauf von 100 Tagen nahezu vollständig besserten [26].

Bezüglich des längerfristigen Verlaufs zeigen auch Daten einer kleinen (*n* = 83) prospektiven Kohorte ohne kardiovaskuläre oder pulmonale Risikofaktoren, dass sich radiologische Veränderungen über die Zeit deutlich besserten [27]: Während 3 Monate nach Hospitalisierung mit COVID-19 78 % der Patient*innen radiografische Veränderungen aufwiesen, zeigten sich nach 9 Monaten noch in 27 % der Fälle radiografische Residuen. Zudem normalisierte sich im Studienkollektiv die körperliche Belastbarkeit gemessen an der 6‑Minuten-Gehstrecke über 12 Monate ebenso wie die Lungenfunktionsparameter FVC und DLCO. Während 3 Monate nach COVID-19 noch 81 % der Kohorte Dyspnoe angaben, war dies nach 12 Monaten nur noch bei 5 % der Kohorte der Fall [27]. Ergänzend hierzu wiesen im Mittel 5 Monate nach COVID-19 persistierend symptomatische Patient*innen in 88 % (59/67) der Fälle eine Schwäche der inspiratorischen Atemmuskulatur mit konsekutiv gesteigertem Atemantrieb auf, was wiederum mit belastungsabhängiger Oxygenierungsstörung, eingeschränkter körperlicher Belastbarkeit und Aktivität sowie höherer gesamtfunktioneller Einschränkung (PCFS) assoziiert war [28].

Das Risiko für eine zwischen 1– 6 Monaten nach COVID-19 auftretende Oxygenierungsstörung ist in der ca. 5 Mio. US-amerikanische Militärveteranen umfassenden Veterans-Health-Administration(VHA-)Kohorte (darunter 73.435 Veteranen nach COVID-19) in Abhängigkeit des initialen Schweregrads 1,5-fach (ambulanter COVID-19-Verlauf) bis ca. 12-fach (intensivmedizinische Betreuung) erhöht [16].

Die Datenlage hinsichtlich Lungenarterienembolien als COVID-19-Folgeerscheinung ist unklarer. Patel et al. berichten von 1 Fall (0,6 %, *n* = 163) binnen 30 Tagen nach COVID-19, wenn nach Entlassung aus dem Krankenhaus keine weitere Thromboseprophylaxe durchgeführt wird [29]. In einer weiteren, kleineren, prospektiven Kohorte (*n* = 33) von nichtinvasiv beatmeten COVID-19-Erkrankten zeigten sich keine thrombembolischen Ereignisse [30]. Auch in unserer eigenen Kohorte zeigte sich bisher kein Fall einer Post-COVID-Lungenarterienembolie (Daten nicht gezeigt). Demgegenüber stehen Daten aus der VHA-Kohorte, die ein etwa 3‑fach erhöhtes Risiko für postakute Lungenarterienembolien nach COVID-19 zeigen (Hazard Ratio, HR: 3,05 (2,36–3,96)), das bei Intensivpatienten sogar auf bis zu 30-fach ansteigt [16].

Hyperinflammationssyndrome, die sich im Anschluss an die akute COVID-19 auch in Form von Alveolitiden fortsetzen können, sind vereinzelt auch bei Erwachsenen in Form des MIS‑A (Multi-Inflammatory Syndrome in Adults) beschrieben [31, 32]. Daten zur Prävalenz oder Inzidenz sind hier bisher nicht verfügbar.

### Kardiovaskuläre Folgeerscheinungen

Differenzialdiagnostisch müssen bei Folgeerscheinungen wie Dyspnoe und thorakalen Beschwerden insbesondere kardiovaskuläre Ursachen in Betracht gezogen werden. Auch intermittierende Tachykardie und Palpitationen sind häufig berichtete Symptome nach COVID-19 [4]. Kontrollierte, prospektive Daten zur Prävalenz spezifischer kardiovaskulärer Folgeerscheinungen sind allerdings kaum verfügbar. Diese sind aber umso wichtiger, da initial in unkontrollierten Querschnittsstudien in der Frühphase nach COVID-19 anhaltende Zeichen myokardialer Inflammation in bis zu 78 % der Fälle im kardialen MRT beschrieben wurden [33].

Eine neuere, hinsichtlich Alter, Geschlecht sowie pulmonaler und kardiovaskulärer Vorerkrankungen/Risikofaktoren kontrollierte Querschnittsstudie aus Cambridge zeigte, dass zum vergleichbaren Zeitpunkt wie in der Puntmann-Studie [33] nur etwa 26 % der Untersuchten Zeichen myokardialer Entzündung im MRT aufweisen [34]. Dennoch zeigte das Cambridge-Kollektiv in der Spiroergometrie Zeichen der kardiorespiratorischen Leistungsminderung. COVID-19-Patient*innen wiesen im Vergleich zur Kontrollgruppe eine niedrigere Sauerstoffaufnahme unter maximaler Belastung (VO2max; 80,5 % vs. 112,7 %), eine reduzierte anaerobe Schwelle (40,7 % vs. 46,8 %) und einen erhöhten VE/VCO2 Slope^1^ als mögliches Zeichen einer Herzinsuffizienz oder pulmonal-vaskulären Belastung auf (33,4 vs. 28,2; [34]).

Ähnliches wurde auch in einem Kollektiv junger Schweizer Armeerekrut*innen (mittleres Alter 21 Jahre) gezeigt. Eine symptomatische SARS-CoV-2-Infektion kann auch bei ansonsten gesunden, jungen Erwachsenen die kardiorespiratorische Leistungsfähigkeit (gemessen an der VO2max) einschränken, während nichtinfizierte Rekrut*innen und asymptomatisch Infizierte diese Einschränkungen nicht aufwiesen [35]. Hierbei ist jedoch zu berücksichtigen, dass VO2max auch von der Ventilation und dem alveolären Gastaustausch abhängt, welche in der Frühphase der COVID-19-Rekonvaleszenz häufig eingeschränkt sind [18, 19, 21]. Eine multizentrische Analyse transthorakaler Echokardiografien 100 Tage nach nichtbeatmungspflichtiger COVID-19 zeigte keine Häufung relevanter Auffälligkeiten [26].

Das Risiko für kardiovaskuläre Akutereignisse nach COVID-19 ist in der VHA-Kohorte gegenüber nichtinfizierten Kontrollpersonen gering erhöht [16]: Dies gilt für Rhythmusstörungen (HR: 1,71 (1,55–1,88)), Myokarditis und Kardiomyopathien (HR: 1,41 (1,19–1,73)) und (nicht näher bezeichnete) Herzinsuffizienz (HR: 1,54 (1,36–1,75)), aber beispielsweise nicht für Myokardinfarkte (HR: 1,04 (0,81–1,35)) oder Asystolie/Kammerflimmern (HR: 1,54 (0,78–3,04); [16]). Keine dieser kardialen Folgeerscheinungen tritt in der VHA-Kohorte im postinfektiösen Intervall zudem häufiger auf, als dies nach saisonaler Influenza der Fall wäre (s. unten, [16]).

Das Risiko für thrombotische oder embolische Ereignisse ist in der VHA-Kohorte gering erhöht (HR: 1,26 (1,02–1,54); [16]). Insgesamt ist die Inzidenz der venösen Thrombembolie nach COVID-19 auf etwa 2,5 % der Fälle zu schätzen [23, 29, 36].

### Zerebrovaskuläre, muskuläre und sensorische Folgeerscheinungen

Das Risiko für zerebrovaskuläre Ereignisse ist nach COVID-19 ebenfalls gering erhöht (HR: 1,41 (1,18–1,69); [16]). Neurologisch sind darüber hinaus parainfektiöse Syndrome wie das Guillain-Barré-Syndrom sowie Hirnnervenausfälle, Myositiden, Plexopathien sowie Enzephalomyelitiden als COVID-19-Folgeerscheinungen berichtet worden [3]. Myopathien sind als COVID-19-Folge deutlich häufiger als nach saisonaler Influenza (HR: 4,70 (3,05–7,24); [16]). Im Übergang zu Folgeerscheinungen aus dem HNO-Bereich werden persistierende Dysgeusie und Dysosmie in bis zu 24 % der Fälle nach akuter COVID-19-assoziierter Riech- und Geschmacksstörung angegeben [3, 4].

### Nephrologische Folgeerscheinungen

Als weitere relevante Folgeerscheinung wiesen in der Wuhan-Kohorte 6 Monate nach Akutinfektion 35 % der Fälle (487/1393) eine eingeschränkte glomeruläre Filtrationsrate auf [23], wobei 13 % der Fälle diese Einschränkung erst im poststationären Verlauf entwickelten [23]. Dies deckt sich mit den VHA-Daten, wonach, abhängig von der initialen Krankheitsschwere von COVID-19, das Risiko für ein akutes Nierenversagen bis zu 5‑fach und für ein chronisches Nierenversagen bis zu 2‑fach erhöht ist [16]. Hierbei scheint es sich um ein SARS-CoV-2-spezifisches Phänomen zu handeln, da das Risiko eines postinfektiösen Nierenversagens nach COVID-19 bei bekanntem renalem Tropismus [37] etwa 1,5-mal höher ist als nach saisonaler Influenza [16]. Der zugrunde liegende Pathomechanismus ist allerdings unklar [38].

### Gastrointestinale Folgeerscheinungen

Das Risiko für das Auftreten gastrointestinaler Folgeerscheinungen wie Diarrhöen, Obstipation oder Refluxerkrankung in Abhängigkeit von der Schwere der Akuterkrankung ist in den ersten 6 Monaten nach COVID-19 bis etwa 5‑fach erhöht [16]. Eine asymptomatische, teilweise monatelange Viruspersistenz im Gastrointestinaltrakt und die daraus resultierende Ausscheidung von Viruspartikeln wurden beschrieben [39]. Zusätzlich wurde in einzelnen Fällen eine Cholangiopathie nach schwerem COVID-19-Verlauf berichtet, die sich histologisch von der sekundär sklerosierenden Cholangitis, wie sie als Folge einer intensivmedizinischen Behandlung bekannt ist, unterscheidet [40].

### Metabolische und endokrinologische Folgeerscheinungen

Auch metabolische und endokrinologische Folgeerscheinungen sind bekannt. Erkrankungen aus dem Formenkreis des metabolischen Syndroms (Adipositas, Hyperlipidämie, Diabetes mellitus) werden in den ersten 6 Monaten nach COVID-19 bis zu 5‑mal häufiger diagnostiziert, wobei unklar ist, inwiefern diese Risikofaktoren eines schweren Akutverlaufs z. B. durch Dokumentationslücken die Statistik verzerren [16].

In der VHA-Kohorte ist das Risiko für hypophysäre Störungen als COVID-19-Folge leicht erhöht (HR: 1,73 (1,10–2,75); [16]), das Risiko für Thyreopathien dagegen nicht [16], was sich mit einer aktuellen Metaanalyse deckt [41].

### Dermatologische Folgeerscheinungen

Dermatologisch stellt sich (passagerer) Haarausfall bei bis zu 25 % der Patient*innen ein [4]. Das Risiko hierfür ist auch in der VHA-Kohorte etwa 1,5- bis 3‑fach erhöht [16]. Ebenso treten mit einem ca. 1,5-fach erhöhten Risiko Hautveränderungen verschiedenster Natur und Ausprägung nach COVID-19 auf [3, 16].

### Inflammation und autoimmunologische Dysregulation

Chronische Hyperinflammation und autoimmunologische Dysregulation könnten COVID-19-Folgeerscheinungen unterhalten [32], insbesondere da in der Akutinfektion bei COVID-19 eine klonale Expansion proinflammatorischer T_H_17-Zellen in der Lunge beschrieben wird, welche mit autoimmunologischen Inflammationssyndromen assoziiert sind [42, 43]. Darüber hinaus können bis zu 5 Monate nach COVID-19 verschiedenste Autoantikörper serologisch nachgewiesen werden [44, 45], deren pathophysiologische, klinische und therapeutische Bedeutung aber noch nicht ausreichend verstanden ist.

### Fatigue als Folgeerscheinung aus somatischer Sicht

Die Ursachen von Fatigue sind multifaktoriell, weswegen auch über das Post-COVID-19-Fatiguesyndrom ausführlich an anderer Stelle in dieser Ausgabe berichtet wird (s. Beitrag von Haller et al.). Aus somatisch-internistischer Sicht sollte eine körperliche Ursache der Beschwerden jedoch immer ausgeschlossen werden (s. unten).

## Spezifität von COVID-19-Folgeerscheinungen

Limitierend in der Interpretation der Spezifität somatischer COVID-19-Folgeerscheinungen ist die heterogene Datenlage. Eine Vielzahl der verfügbaren Studien hat kleine Kollektive untersucht, meist im Design von Querschnittsstudien. Kontrollgruppen fehlen häufig. Sollten Kontrollgruppen mituntersucht sein, dann meist nicht in Form von Infektionskontrollen (z. B. saisonale Influenza als Referenzerkrankung). In Kombination mit fehlenden individuellen Vorbefunden bzgl. der untersuchten funktionellen Parameter könnte so ein Bias eingeführt werden. Ob es sich bei COVID-19-Folgeerscheinungen um Paraphänomene in der Folge eines (schweren) generalisierten Virusinfekts handelt, ist daher weiter Gegenstand der wissenschaftlichen Debatte.

Die verfügbaren Daten zeigen allerdings, dass der akute Verlauf von COVID-19 bei Hospitalisierung deutlich schwerer verläuft als die saisonale Influenza [46]. Auch hinsichtlich organbezogener Folgeerscheinungen ist in der VHA-Kohorte das Risiko von Myopathien und thrombembolischen Ereignissen, inkl. Lungenarterienembolie, nach COVID-19 höher als nach saisonaler Influenza. Das Risiko für z. B. Myokarditis, Herzinsuffizienz oder Herzrhythmusstörungen nach Hospitalisierung unterschied sich dagegen nach COVID-19 nicht von der saisonalen Influenza. [16]. Insgesamt zeigt sich aber nach COVID-19 ein erhöhtes adjustiertes Risiko für Folgen der intensivmedizinischen Behandlungspflichtigkeit im Vergleich zur Influenza [16]. Die VHA-Daten deuten aber darauf hin, dass die meisten Folgeerscheinungen eher Ausdruck einer schweren akuten Viruserkrankung sein könnten, wobei gerade die Identifikation COVID-19-spezifischer Folgeerscheinungen weiter von hohem wissenschaftlichen Interesse ist.

## Diagnostikempfehlungen

**Figure 2:**
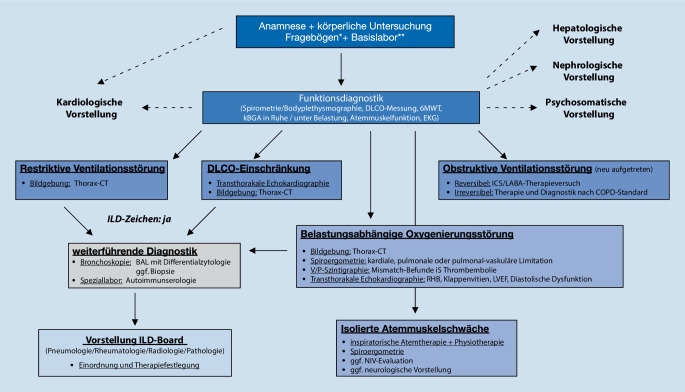

Die Diagnostik von organbezogenen COVID-19-Folgeerscheinungen sollte basierend auf den Leitsymptomen erfolgen. Die Komplexität in der Diagnostik entsteht jedoch durch die häufig unspezifische Symptomatik der Patient*innen. Daher bietet sich zunächst eine Basisdiagnostik an, welche dann modular erweitert werden kann [3]. Exemplarisch ist solch ein modularer Diagnosealgorithmus aus unserer pneumologischen COVID-19-Nachsorge in Abb. 2 dargestellt.

## Therapieoptionen

Derzeit kann keine generelle, medikamentöse Therapie bei COVID-19-Folgen empfohlen werden. Therapie- und Rehabilitationsmaßnahmen richten sich nach Art und Ausmaß der spezifischen COVID-19-Folgeerscheinung [47]. Während beispielsweise ein COVID-19-assoziierter Haarverlust keiner Therapie bedarf [3], sollte bei Post-COVID-19-Alveolitis nach entsprechender pneumologischer Diagnostik eine Steroidstoßtherapie erwogen werden [25]. Auf COVID-19-Folgen zugeschnittene Rehabilitationsmaßnahmen in spezialisierten Zentren können nicht nur lungenfunktionelle Marker, sondern auch allgemeine Muskelkraft, kognitive und weitere psychische Parameter verbessern [47, 48]. Darüber hinaus scheint für bestimmte Patient*innen inspiratorisches Atemmuskeltraining empfehlenswert [28, 49]. SARS-CoV-2-Vakzine können das Risiko für das Auftreten von COVID-19-Folgeerscheinungen nach Durchbruchsinfektionen [50] und die Symptomlast bei bestehenden Folgeerscheinungen in etwa einem Viertel der Fälle (23,9 %) reduzieren [51].

## Fazit

COVID-19-Folgeerscheinungen sind häufig und vielgestaltig. COVID-19-Folgeerscheinungen, die länger als 4 Wochen anhalten oder später neu auftreten, werden als „Long-COVID“ bezeichnet. Organbezogene COVID-19-Folgeerscheinungen treten häufiger nach schweren Akutverläufen auf. Ersten Daten zufolge bessern sich die meisten organbezogenen COVID-19-Folgeerscheinungen über die Zeit in klinisch relevantem Ausmaß. Die SARS-CoV-2-Spezifität der Folgeerscheinungen ist nicht abschließend geklärt. Therapie und Rehabilitationsmaßnahmen richten sich nach Art, Umfang und Ursache der jeweiligen Folgeerscheinung. Allgemeinmedikamentöse oder spezifische Therapieoptionen bestehen für Long-COVID bisher nicht. Aufgrund der Mannigfaltigkeit der Symptome und Beschwerden stellen die adäquate Diagnostik und Versorgung von COVID-19-Folgeerscheinungen eine interdisziplinäre Herausforderung dar und bedürfen idealerweise einer zentralisierten Gatekeeper-Struktur, mit raschem Zugriff auf Spezialressourcen, soweit bzw. sobald diese erforderlich werden.

## Footnotes

1 Atemeffizienz als Anstiegssteilheit der Regressionsgeraden zwischen Ventilation und Kohlendioxidabgabe.
